# How many lives must be lost before we consider SUDEP counselling an obligation rather than a choice? The need for public policies

**DOI:** 10.1111/ene.16407

**Published:** 2024-07-06

**Authors:** Kette Valente, Susanne Knake

**Affiliations:** ^1^ University of São Paulo Faculty of Medicine São Paulo Brazil; ^2^ Epilepsy Center Hessia, Department of Neurology Philipps‐University Marburg Germany

Governments will always play a huge part in solving big problems. They set public policy and are uniquely able to provide the resources to ensure solutions reach everyone who needs them. *Bill Gates*

Sudden unexpected death in epilepsy (SUDEP) is a leading cause of premature death in people with epilepsy (PWE). Based on a review of 12 studies, the estimated incidence is 0.22 per 1000 person‐years in children and 1.2 per 1000 person‐years in adults [1]. SUDEP is listed in the 11th edition of the International Classification of Diseases for Mortality and Morbidity Statistics (ICD‐11/MH15), being defined as sudden death in a person with epilepsy that occurs under benign circumstances and in the absence of a known structural cause of death, thereby ruling out drowning, fatal injuries, poisoning, or other internal or external factors [2].

Over the last decades, research has increased exponentially, emphasizing SUDEP and its mechanisms, risk factors, and potential biomarkers. Despite the growing knowledge and some paradigm shifts, communication, and counselling remain a barrier.

A global survey documented heterogeneity in counselling attitudes on SUDEP, with the lowest rates in Africa and the highest rates in North and South America. In addition, healthcare providers (HCPs) with higher levels of education about epilepsy (epilepsy fellowship and working in the academic setting) felt more comfortable discussing SUDEP [3].

In this issue of the *European Journal of Neurology*, Watkins et al. [4] surveyed HCPs in the UK (197 respondents) and Norway (112 respondents), documenting their attitudes toward SUDEP counselling. Despite socio‐demographic similarities, UK HCPs gave importance to SUDEP, and referred and had access to bereavement support. Consequently, HCPs in the UK communicate and inform about SUDEP more often. The attitudes about communication and ensuring that patients understood SUDEP were fundamentally influenced by nurses. Norway HCPs are more likely to refrain from discussing SUDEP with their patients.

The disparities between these two high‐income countries are intriguing since Norway is a country with high levels of literacy and a high‐ranked healthcare system. Several historical and cultural factors may be postulated to explain these findings.

One possible aspect is that the UK has a leadership in SUDEP awareness and advocacy. The landmark audit of epilepsy‐related deaths, released in the UK on 20 May, which identified that 40% of all SUDEP deaths could have been avoided, had a strong impact locally and globally [5]. These unsettling data led to an essential editorial for a wake‐up call about sudden death [6]. As a result, SUDEP has been included in epilepsy guidelines for health professionals in the National Institute for Health and Care Excellence, which was updated in 2022 [7]. In addition, the proactive SUDEP Action has played a significant role in awareness and provided a relevant path for communication between HCPs and patients/families.

Although Watkins et al. identified best practices in the UK, only 52% of HCPs discussed SUDEP with ‘new patients’, and 73% discussed SUDEP if they identified a change in ‘risk’. In addition, 26% of HCPs in Norway rarely or never discuss SUDEP. A limitation of the current survey is the lack of information about who is defined as ‘at risk’ and what is considered ‘risk modification’.

In general, HCPs identify poor antiseizure medication adherence, frequent tonic–clonic seizures or drug‐resistant epilepsy as risk factors [3]. PWE, mainly adults with severe epilepsy who are receiving polytherapy, are more likely to receive counselling. Indeed, PWE with uncontrolled seizures are at higher risk than well‐controlled patients. Although there is no consensus about the timing to provide information, risk prediction is complex and not easily made on daily bases by clinicians. Potential risk prediction tools include several clinical predictor variables [8].

The occurrence of tonic–clonic seizures, generalized or bilateral, in the previous year, followed by sleeping or living alone, increases the risk of SUDEP. These two factors combined are associated with an odds ratio for SUDEP of 67 [9].

Medication adherence, avoiding substance abuse, and not living alone or sleeping under supervision are decisions made by families and patients who might prevent SUDEP if they are adequately informed.

One may ask if counselling is an option. This question may be seen from different angles, leading to the same response. First, guidelines, such as the American Academy of Neurology [3] and the National Institute of Clinical Excellence [7], strongly advise information disclosure about SUDEP to all patients. Second, current evidence points to modifiable risk factors when information is provided.

Finally, although patient distress and anxiety have been identified as a reason for not providing counselling, only PWE and their caregivers have the right to decide what information must be disclosed and what actions to take, such as refresher training in basic life support. Bereaved relatives and PWE have already responded with a solid and clear message. Relatives and PWE opted for information on SUDEP at the time of, or shortly following, the diagnosis of epilepsy. Neurologists were identified as the healthcare providers who should discuss SUDEP with patients during a face‐to‐face encounter, supplemented with written information. It was identified that, when discussing SUDEP, emphasis should be on risk factors, possible preventive strategies and the rarity of incidence [10, 11].

The paternalistic approach may no longer be accepted. Public policies, education, training, and support may provide the path to what PWE, not physicians, consider optimal practices to hopefully reduce the risk of SUDEP.

## AUTHOR CONTRIBUTIONS

**Kette Valente:** Conceptualization; writing – original draft; validation; writing – review and editing. **Susanne Knake:** Validation; writing – review and editing.

## CONFLICT OF INTEREST STATEMENT

None.

## Data Availability

Data sharing is not applicable to this article as no new data were created or analyzed in this study.
